# Supplementary material for: Clinical data-based modeling of IVF live birth outcome and its application
Source: Reprod Biol Endocrinol. 2024 Jul 8;22:76. doi: 10.1186/s12958-024-01253-3 (PMC11229224; doi:10.1186/s12958-024-01253-3)
Supplement: Supplementary file 1 — Supplementary Material 1 [file 12958_2024_1253_MOESM1_ESM.docx]

**Clinical data-based modeling of IVF live birth outcome and its application**

Liu Liu^1^, Hua Liang^1^, Jing Yang^2^, Fujin Shen^1*^^[[1]](#footnote-1)^#, Jiao Chen^2^, and Liangfei Ao^3*^^[[2]](#footnote-2)^#

^1^ Department of Obstetrics and Gynecology, Renmin Hospital of Wuhan University, Wuhan, China

^2^ Reproductive Medicine Center, Renmin Hospital of Wuhan University, Wuhan, China

^3^ Wuhan Jinxin Gynecology and Obstetrics Hospital of Integrative Medicine, Wuhan, Hubei, China

^*^Correspondence: (Fujin Shen) shenfj_rmh@outlook.com and (Liangfei Ao) aoliangfei@whu.edu.cn

**Abstract**

**Background:** The low live birth rate and difficult decision-making of the in vitro fertilization (IVF) treatment regimen bring great trouble to patients and clinicians. Based on the retrospective clinical data of patients undergoing the IVF cycle, this study aims to establish classification models for predicting live birth outcome (LBO) with machine learning methods. **Methods:** The historical data of a total of 1405 patients undergoing IVF cycle were first collected and then analyzed by univariate and multivariate analysis. The statistically significant factors were identified and taken as input to build the artificial neural network (ANN) model and supporting vector machine (SVM) model for predicting the LBO. By comparing the model performance, the one with better results was selected as the final prediction model and applied in real clinical applications. **Results:** Univariate and multivariate analysis shows that 7 factors were closely related to the LBO (with *P* < 0.05): Age, ovarian sensitivity index (OSI), controlled ovarian stimulation (COS) treatment regimen, Gn starting dose, endometrial thickness on human chorionic gonadotrophin (HCG) day, Progesterone (P) value on HCG day, and embryo transfer strategy. By taking the 7 factors as input, the ANN-based and SVM-based LBO models were established, yielding good prediction performance. Compared with the ANN model, the SVM model performs much better and was selected as the final model for the LBO prediction. In real clinical applications, the proposed ANN-based LBO model can predict the LBO with good performance and recommend the embryo transfer strategy of potential good LBO. **Conclusions:** The proposed model involving all essential IVF treatment factors can accurately predict LBO. It can provide objective and scientific assistance to clinicians for customizing the IVF treatment strategy like the embryo transfer strategy.

**Keywords:** IVF; machine learning; live birth outcome; embryo transfer strategy, clinical decision support

**1. Introduction**

As one of the most effective infertility treatments, assisted reproductive technology (ART) is becoming increasingly advanced and widely utilized due to continuous improvements in essential technologies such as controlled ovarian stimulation (COS), ultrasound-guided oocyte collection, sperm processing, embryo culture and transfer, pre-embryo transfer genetic diagnosis, etc. Despite significant advancements, the success rate of In vitro fertilization (IVF) cycle seems to have reached a plateau: currently, the clinical success rate still hovers at 40-50% with the final live birth rate of around 30% [1]. Given that the primary objective of IVF treatment is to attain live birth, the current situation is far from ideal. The low success rate, necessity for repeated cycles, costly treatments, and complex procedures impose a significant financial and emotional burden on infertile couples. If clinicians are able to make an accurate and reliable prediction of the live birth outcome (LBO) prior to the IVF cycle and adjust the treatment strategies accordingly, improved LBO could be achieved, which would hold great significance for both clinicians and patients.

Several factors have been identified as potential influencers of the final pregnancy outcome, such as basic clinical characteristics, COS strategies, embryo transfer-related details, and so forth [2]. Van Loendersloot et al. [3] developed a pregnancy rate prediction model based on 13 indicators including the female age, duration of subfertility, previous ongoing pregnancy, male subfertility, diminished ovarian reserve, endometriosis, basal follicle stimulating hormone (bFSH), number of failed IVF cycles, fertilization, number of embryos, mean morphological score per Day 3 embryo, presence of 8-cell embryos on Day 3 and presence of morulae on Day 3. Nevertheless, we believe that the prediction of LBO deserves more attention than that of the pregnancy rate. On this particular research topic, a retrospective cohort study conducted by Metello et al. [4] showed that the age of the patient, anti-Müllerian hormone (AMH), antral follicle count (AFC), and infertility factors are significant determinants of LBO. Additionally, a multi-center big data study conducted by Wen et al. [5] confirmed that female age, cycle number, female body mass index (BMI), male factor, ovulation disorder, and endometrial thickness are important predictors of LBO. The current prediction models have only focused a limited number of predictors before the IVF cycle and lacked consistent and reliable standards, which limits their application in real clinical practice. For a complete IVF cycle, there are numerous factors influencing the LBO, especially after oocyte retrieval (such as the embryo transfer strategy). Therefore, it is highly meaningful to comprehensively evaluate the influencing factors in the IVF cycle (including post-oocyte retrieving factors) and establish a comprehensive and accurate prediction model for LBO.

Based on historical clinical information of patients and machine learning methods, this research aims to develop a prediction model for LBO (i.e., a classification model of live birth or not), which fully considers representative indicators of patients during IVF-ET cycle, such as basic clinical characteristics, COS strategies, ovarian responsiveness to gonadotropin (Gn), embryo transfer strategy, etc. The proposed model is then utilized for predicting the LBO as well as optimizing the embryo transfer strategy in real clinical practice. The establishment of the LBO model can play a positive role in optimizing treatment plans, reducing short-term and long-term risks of IVF treatment, and improving LBO. Additionally, it can provide a scientific and objective assessment of the outcome of IVF treatment, alleviate patient's psychological burden, and increase treatment confidence and patient’s compliance during the process.

**2. Methods**

**2.1 Study design and participants**

Patients undergoing IVF/Intracytoplasmic sperm injection (ICSI) cycle in the Reproductive Center of Renmin Hospital of Wuhan University were enrolled in our study, and each patient is transferred with one or two embryos in each fresh cycle. Exclusion criteria comprised: (1) pulmonary tuberculosis; (2) serious medical diseases such as hypertension, diabetes, liver, and kidney diseases; (3) uterine malformation, intrauterine adhesion and hydrosalpinx; (4) oocyte donation cycles or natural cycles; (5) Progestin-primed ovarian stimulation (PPOS), luteal phase stimulation or micro-stimulation program; (6) chromosomal abnormalities in infertile couples. A total of 1405 women's clinical data were included in developing the LBO prediction model.

IVF patients underwent COS and transvaginal oocyte retrieval following human chorionic gonadotropin (HCG) trigger when one or two dominant follicles reached 18 mm in diameter. The selected sperm and egg was fertilized to form embryos. One or two embryos were cultured and transferred at either cleavage stage (2-3 days after oocyte collection) or blastocyst stage (5-6 days after oocyte collection). Serum HCG test and B-ultrasonography were conducted 14 and 30 days respectively post-transfer to confirm pregnancy. Live birth was defined as the delivery of a fetus alive at 28 weeks gestation, who remained alive for at least one month.

To build the LBO model, 16 related factors were analyzed initially in our study, including 5 basic clinical characteristics (age, BMI, infertility type, infertility duration, infertility cause); and 11 clinical cycle indexes (COS treatment regimen, Gn starting dose, Ovarian sensitivity index (OSI), E_2_ level on HCG day, Progesterone (P) value on HCG day, luteinizing hormone (LH) value on HCG day, endometrial thickness on HCG day; pronuclei (2PN) number, transferable embryos number, high-quality embryos number, embryo transfer strategy (the stage and embryos transferred number)).

The proposed research is outlined in Figure 1.


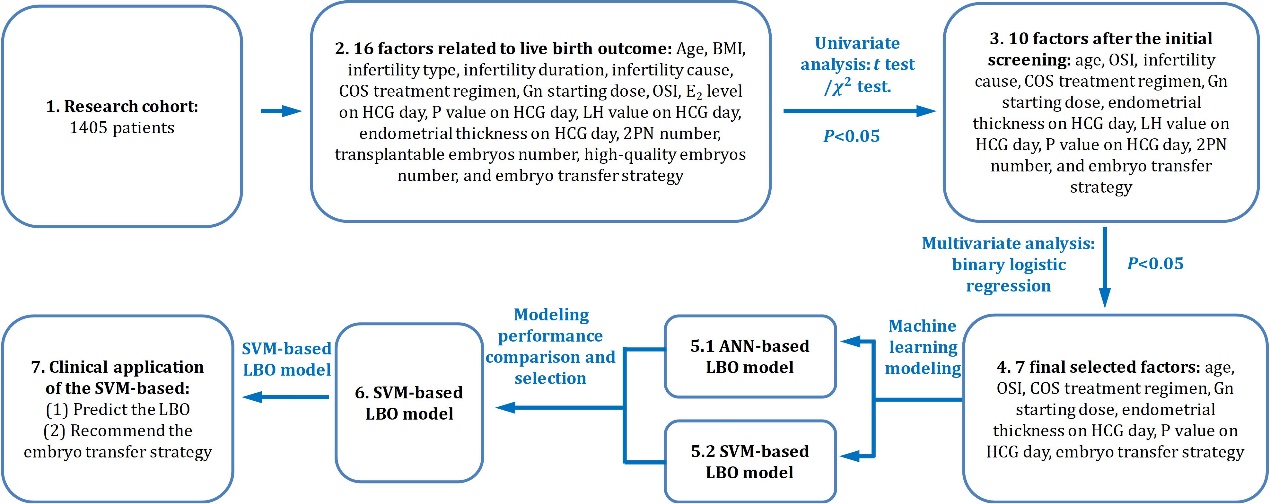


**Figure 1** Overview of the proposed research. (1)~(2) Collect data of patients and find the factors related to the LBO; (3) Conduct univariate analysis to determine the significant influencing factors of LBO initially; (4) Conduct multivariate analysis to determine the influencing factors of LBO further; (5) Based on machine learning method, the ANN-based LBO model and SVM-based LBO model are built; (6) The prediction performance of the two models are compared, and the one, i.e., the SVM-based LBO model, that has better modeling performance is selected. (7) 81 new patients were involved in the clinical application of the proposed model to verify the prediction performance and the function of recommendation of the embryo transfer strategy.

**2.2 Machine learning methods**

The LBO prediction model aims to, by taking the clinical information of patients as input, output the result of live birth or not. Therefore, it is a classification model. This study uses two typical classification models, i.e., artificial neural network (ANN) and support vector machines (SVM) to build the LBO prediction model. The corresponding two models are defined as the ANN-based LBO model and the SVM-based LBO model.

**2.3.1 Artificial neural network model**

By choosing appropriate ANN hyperparameters, ANN, in theory, can approximate any linear and nonlinear function. The dataset is randomly divided into a training set (70%), a validation set (17%), and a test set (13%). These sets are respectively used for calibrating and optimizing the parameters of the ANN, adjusting the hyperparameters and complexity of the model, and testing the generalization ability of the trained ANN model. Note that the sample division for the three sets is determined via a trial-and-error method: we have tried dozens of combinations for the percentage of training, validation, and test set, and the one with the best prediction performance is our final selection. During training, the Polak-Ribiére conjugate gradient algorithm is employed to update the parameters of the neural network, with the iteration termination conditions being: the maximum number of iterations is 2000, the minimum gradient is ${1\times10}^{-10}$, or the minimum iteration step size is ${1\times10}^{-6}$. Since the proposed model is a classification model, cross entropy function is selected as the loss function.

**2.3.2 Support vector machines (SVM) model**

SVM is also a powerful classification model in machine learning. When training the SVM model, the data is randomly divided into a training set (80%) and a test set (20%), with the main parameters set as follows: SVM kernel function: Gaussian function; kernel scale parameters: automatically selected; optimization algorithm: ISDA iterative single data algorithm. Other parameters of the SVM model (such as the number of cross-folds, tolerance of gradient differences, maximum number of numerical optimization iterations, etc.) are automatically selected and optimized using the OptimizeHyperparameters function of the SVM algorithm.

**2.3.3 Evaluation indicators of the prediction model**

The following indicators of classification models can express the modeling performance of the proposed ANN-based and SVM-based LBO:

(1) Graphic indicators: receiver operating characteristic (ROC) curve and area under curve (AUC) value. Among them, the closer the ROC curve is toward the point (0,1), i.e., the further it deviates from the 45-degree diagonal to the upper left corner, the larger the area of the AUC, and the better performance of the classification model.

(2) Quantitative indicators: precision, sensitivity (also called recall), accuracy, and F1 score. The greater the precision, sensitivity, accuracy and F1 score, the better the performance of the prediction model.


**2.3 Clinical application and validation of the model**

The clinical application of the proposed model is applied to the incoming patients after the model has been built. With the same screening criterion as these for building the mode, we select 82 patients who underwent IVF-ET treatment at the Reproductive Center of Renmin Hospital of Wuhan University, to whom we apply the proposed model for predicting the LBO and customizing the embryo transfer strategy.

**2.4 Statistical and machine learning model**

Univariate and multivariate analyses were applied to determine whether the 16 factors, as previously described, had statistically significant effects on the LBO. The selected meaningful influencing factors (*P*＜0.05) were taken as input to establish the machine learning model of LBO. Data processing and correlation analysis are completed in IBM SPSS Statistics 24. In the univariate analysis, continuous and categorical variables were analyzed by $t$-test and $\chi^{2}$-test, respectively, while the multivariate analysis was conducted by binary logistic regression. A two-sided test was performed, with *P*<0.05 considered significant.

The ANN and SVM machine learning method is programmed and implemented in MATLAB R2021a. The software prototype “Decision Support System of IVF–Embryo transfer strategy recommendation”, as shown in Figure 4, is developed in Visual Studio 2019 and QT 6.0.

**3. Results**

**3.1 Univariate analysis of factors influencing live birth outcomes**

The 1405 patients were divided into a live birth group (592 patients) and a non-live birth group (813 patients), as listed in Table 1. Univariate analysis revealed that 10 out of the 16 potential influencing factors exhibited statistical significance on the outcome of live birth: age (*P*<0.001), OSI (*P*=0.003), infertility cause (*P*=0.048), COS treatment regimen (*P*<0.001), Gn starting dose (*P*=0.001), endometrial thickness on HCG day (*P*=0.007), LH value on HCG day (*P*<0.001), P value on HCG day (*P*=0.032), 2PN number (*P*=0.049) and embryo transfer strategy (*P*<0.001). The age, P value of HCG day, and LH value of HCG day in the live birth group were significantly lower than those in the non-live birth group (*P*＜0.05); and OSI, Gn starting dose, endometrial thickness on HCG day and 2PN number of the live birth group were significantly higher than those of non-live birth group (*P*＜0.05). However, there were no statistically significant differences in infertility type, infertility duration, BMI, E_2_ level on HCG day, transferable embryo number, and high-quality embryo number between the live birth group and the non-live birth group (*P*>0.05).

**Table 1** Demographic information and univariate analysis results of the 16 influencing factors of the LBO model

| Characteristics | | Live birth group （592） | Non-live birth group（813） | Statistic | *P* |
| --- | --- | --- | --- | --- | --- |
| **Age** |  | **30.55（3.52）** | **32.17（4.21）** | **13.985^*^** | **<0.001** |
| Infertility type （%） | Primary infertility | 330（55.74） | 430（52.89） | 1.123^**^ | 0.289 |
|  | Secondary infertility | 262（44.26） | 383（47.11） |  |  |
| Infertility duration |  | 3.87（2.65） | 3.33（2.43） | 2.536^*^ | 0.112 |
| BMI |  | 22.32（3.22） | 22.23（3.10） | 1.508^*^ | 0.220 |
| **OSI** |  | **5.75（3.49）** | **5.52（3.85）** | **8.848^*^** | **0.003** |
| **Infertility causes** | Pelvic and fallopian tube factors | **270（45.61）** | **341（41.94）** | **62.019^**^** | **0.048** |
|  | PCOS ovulatory obstacle | **47（7.94）** | **33（4.06）** |  |  |
|  | Poor ovarian reserve | **37（6.25）** | **71（8.73）** |  |  |
|  | Endometriosis | **31（5.24）** | **32（3.94）** |  |  |
|  | Multiple factors | **40（6.76）** | **162（19.93）** |  |  |
|  | Others | **167（28.21）** | **174（21.40）** |  |  |
| **COS treatment regimen** | GnRH agonist long protocol | **247（41.72）** | **222（27.31）** | **57.254^**^** | **<0.001** |
|  | Ultra-long GnRH agonist protocol | **251（42.40）** | **332（39.95）** |  |  |
|  | GnRH antagonist protocol | **94（15.88）** | **259（31.17）** |  |  |
| **Gn starting dose** |  | **188.75 （60.99）** | **177.87（70.97）** | **11.668^*^** | **0.001** |
| **Endometrial thickness on HCG day** |  | **12.22（2.41）** | **10.40（2.39）** | **2.552^*^** | **0.007** |
| E_2_ level on HCG day |  | 2976.78 （1398.96） | 2521.44（1294.69） | 0.650^*^ | 0.420 |
| **LH value on HCG day** |  | **1.28（1.39）** | **1.72（2.07）** | **18.077^*^** | **<0.001** |
| **P value on HCG day** |  | **0.75（0.35）** | **0.99（0.33）** | **0.480^*^** | **0.032** |
| **2 PN number** |  | **6.70（3.33）** | **6.05（3.64）** | **3.582^*^** | **0.049** |
| Transferable embryos number |  | 4.81（2.59） | 4.01（2.50） | 4.369^*^ | 0.067 |
| High-quality embryos number |  | 4.24（2.71） | 3.26（2.77） | 0.432^*^ | 0.511 |
| **Embryo transfer strategy** | **1 embryo at cleavage stage** | **10（1.69）** | **55 （6.77）** | **72.484^**^** | **<0.001** |
|  | **2 embryos at cleavage stage** | **401（67.74）** | **464（55.07）** |  |  |
|  | **1 embryo at blastocyst stage** | **62（10.47）** | **194（23.86）** |  |  |
|  | **2 embryos at blastocyst stage** | **119（20.10）** | **100（12.30）** |  |  |

The OSI is calculated as the number of retrieved oocytes (among which the percentage of mature oocytes is more than 95%)/total gonadotropin dose × 1000.

Superscript ^*^ represents $t$-test while ^**^ represents $\chi^{2}$-test.

Values are expressed as quantity (percentage) or mean (variance).

The bold type indicates the significant influencing factors with *P* < 0.05.

**3.2 Multivariate analysis of factors influencing live birth outcomes**

Ten factors selected in univariate analysis (age, OSI, infertility cause, COS treatment regimen, Gn starting dose, endometrial thickness on HCG day, LH value on HCG day, P value on HCG day, 2PN number, embryo transfer strategy) were further included in binary logistic regression for multivariate analysis. The results are presented in Table 2. It shows that age (*P*<0.001), OSI (*P*=0.027), COS treatment regimen (*P*≤0.028), Gn starting dose (*P*<0.001), endometrial thickness on HCG day (*P*<0.001), P value on HCG day (*P*=0.023), embryo transfer strategy (*P*≤0.045) were significant correlated with LBO (*P*<0.05). The remaining three factors (infertility causes, LH value on HCG day, and 2PN number) have no significance (*P* > 0.05), and therefore were excluded in the subsequent modeling of LBO process.

**Table 2** Multivariate analysis results of the 10 initial screened impact factors

| Characteristics | | B (Partial regression parameter) | S.E. | Wals | OR (Odds ratio) | 95% CI | *P* value |
| --- | --- | --- | --- | --- | --- | --- | --- |
| **Age** |  | **-0.122** | **0.018** | **47.327** | **0.885** | **0.855-0.916** | **<0.001** |
| **OSI** |  | **0.110** | **0.000** | **10.275** | **0.912** | **0.526-0.949** | **0.027** |
| Infertility causes | Pelvic and fallopian tube factors | Ref |  |  |  |  |  |
|  | PCOS ovulatory obstacle | 0.828 | 0.266 | 9.705 | 2.289 | 1.359-3.854 | 0.051 |
|  | Poor ovarian reserve | -0.093 | 0.254 | 0.135 | 0.911 | 0.554-1.499 | 0.714 |
|  | Endometriosis | 0.150 | 0.291 | 0.265 | 1.162 | 0.657-2.056 | 0.606 |
|  | Multiple factors | -0.819 | 0.213 | 14.733 | 0.441 | 0.290-1.670 | 0.513 |
|  | Others | 0.347 | 0.151 | 5.313 | 1.145 | 1.053-1.902 | 0.053 |
| **COS treatment regimen** | GnRH agonist long protocol | **Ref** |  |  |  |  |  |
|  | Ultra-long GnRH agonist protocol | **-0.326** | **0.149** | **4.820** | **0.722** | **0.593-0.966** | **0.028** |
|  | GnRH antagonist protocol | **-1.067** | **0.182** | **34.230** | **0.344** | **0.241-0.492** | **<0.001** |
| **Gn starting dose** |  | **0.007** | **0.001** | **36.412** | **1.007** | **1.005-1.009** | **<0.001** |
| **Endometrial thickness on HCG day** |  | **0.110** | **0.026** | **18.453** | **1.117** | **1.062-1.174** | **<0.001** |
| LH value on HCG day |  | -0.044 | 0.048 | 0.832 | 0.957 | 0.870-1.052 | 0.362 |
| **P value on HCG day** |  | **-0.310** | **0.176** | **2.974** | **0.734** | **0.520-0.735** | **0.023** |
| 2 PN number |  | 0.026 | 0.023 | 1.352 | 1.027 | 0.982-1.073 | 0.245 |
| **Embryo transfer strategy** | **1 embryo at the cleavage stage** | **Ref** |  |  |  |  |  |
|  | **2 embryos at the cleavage stage** | **1.032** | **0.382** | **7.295** | **2.807** | **0.146-0.742** | **0.007** |
|  | **1 embryo at blastocyst stage** | **0.109** | **0.419** | **0.068** | **0.897** | **0.539-1.037** | **0.045** |
|  | **2 embryos at the blastocyst stage** | **1.111** | **0.415** | **7.174** | **3.036** | **1.347-6.845** | **<0.001** |
| Constant |  | 0.613 | 0.784 | 0.612 | 1.846 | / | 0.034 |

The bold type indicates the significant influencing factors with *P* < 0.05.

**3.3 Live birth outcome model based on machine learning**

Given the 7 final selected impact factors from multivariate analysis, the proposed ANN-based and SVM-based LBO model can be built with the input and output being:

7 inputs: Age, OSI, COS treatment regimen, Gn starting dose, endometrial thickness on HCG day, P value on HCG day, and embryo transfer strategy.

1 output: LBO (whether live birth is achieved or not).

**3.3.1 Modeling results of ANN-based LBO model**

The total 1405 samples were randomly divided into a training set (979 cases), a verification set (243 cases), and a test set (183 cases). To effectively train the model, the samples in the three sets are randomly chosen from the total dataset, and each sample can be randomly chosen only once when constructing the three sets. In this way, the model can be trained with good prediction performance and generalization. The number of nodes and hidden layers of the ANN model was determined by trial and error: the ANN-based LBO model in this work has 2 hidden layers containing 5 and 3 nodes, respectively.

The modeling performance of the proposed ANN-based LBO is shown in Figure 2. The ROC curves of the training set, validation set, and test set are shown in Figure 2(a), (b), and (c), respectively, with the AUC being 0.726, 0.719, and 0.701. The precision, sensitivity, accuracy, and F1 score is listed in Table 3.


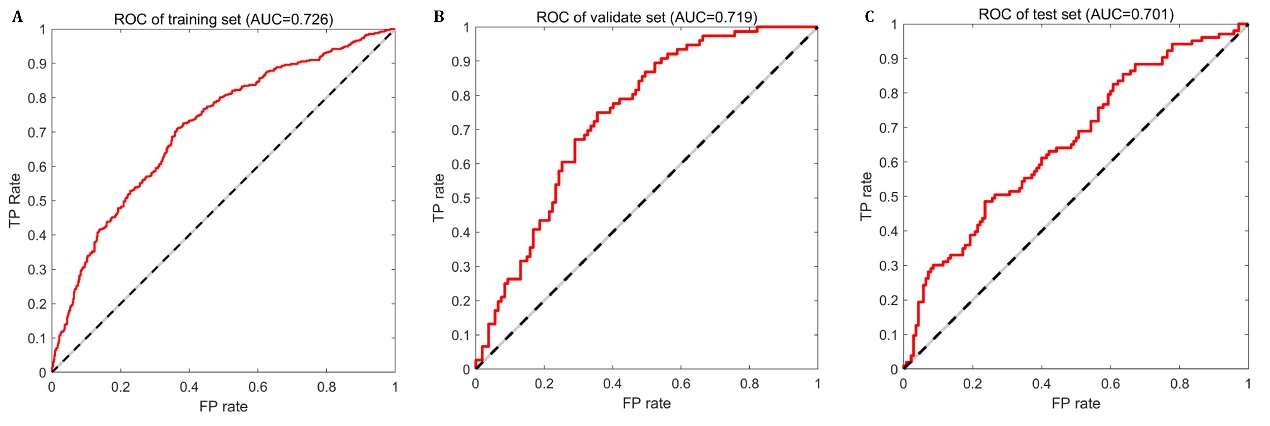


**Figure 2** ROC curves of ANN-based LBO model. (A) the training set; (B) the validation set; (C) the test set.

**Table 3** Modeling performance of the proposed ANN-based LBO model

|  | AUC | Precision（%） | Sensitivity（%） | Accuracy（%） | F1 Score（%） |
| --- | --- | --- | --- | --- | --- |
| Training set | 0.726 | 67.23 | 67.72 | 72.52 | 67.47 |
| Validation set | 0.719 | 65.09 | 66.99 | 70.78 | 66.03 |
| Test set | 0.701 | 62.96 | 68.92 | 71.04 | 65.81 |

The overall prediction performance of the proposed model on the training set is a litter better than that on the validation and test sets. This is because the parameters of the ANN model itself are calibrated and optimized from the training set. In addition, there is not much difference between the performance of the two, indicating that the established ANN-based LBO model has good generalization ability.

**3.3.2 Modeling results of SVM-based LBO model**

Unlike the ANN model, the SVM model only needs to divide the samples into two groups: training set (1124) and test set (281). The two sets are also generated randomly, like those for the ANN model. For both the training set and test set, the established SVM-based LBO model has good classification performance, with the two ROC curves shown in Figure 3 and AUCs being 0.912 and 0.854, respectively. The precision, sensitivity, accuracy, and F1 score are listed in Table 4.


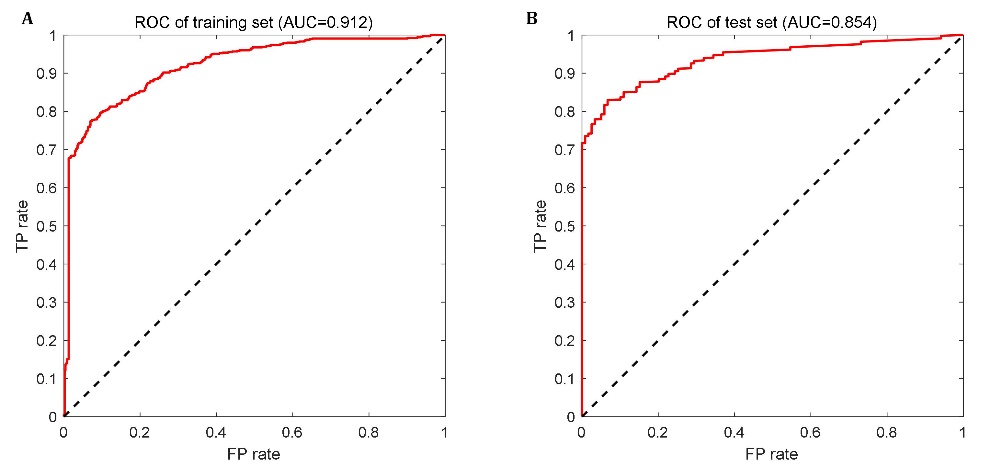


**Figure 3** ROC curves of SVM-based LBO model. (A) the training set; (B) the test set.

Compared with the test set, the training set has better performance except for sensitivity. This is because the parameters of the SVM model itself are determined based on the training set, so it has a better classification effect on the training set. In addition, for the test set itself, the SVM model's AUC=0.854 and F1 score=77.18% for its classification results, indicating that the established model has good generalization ability, facilitating its further clinical application and verification.

**Table 4** Modeling performance of the proposed SVM-based LBO model

|  | AUC | Precision（%） | Sensitivity（%） | Accuracy（%） | F1 Score（%） |
| --- | --- | --- | --- | --- | --- |
| Training set | 0.912 | 86.41 | 75.58 | 84.78 | 80.63 |
| Test set | 0.854 | 77.50 | 76.86 | 80.43 | 77.18 |

**3.3.3 Comparison and selection of the final LBO model**

To select the final model for predicting the LBO of the IVF-ET cycle, the modeling performance of the proposed two models is compared. Regardless of the training set or the test set, the modeling evaluation indicators of SVM-based model is significantly better these of the ANN model: training set (AUC: 0.912 vs. 0.726; precision: 86.41% vs. 67.23%; sensitivity: 75.58% vs. 67.72%; accuracy: 84.78% vs. 75.52%; F1-score: 80.63% vs. 67.47%); test set (AUC: 0.854 vs. 0.701; precision: 77.50% vs. 62.96%; sensitivity: 76.86% vs. 68.92%; accuracy: 80.43% vs. 71.04%; F1-score: 77.18% vs. 65.81%). Therefore, the SVM-based LBO model is selected as the final model for predicting the LBO of patients under the IVF-ET cycle.


**3.4 Validation and clinical application of the proposed SVM-based LBO model**

**3.4.1 Validation of the proposed SVM-based LBO model**

Based on the proposed SVM-based LBO model, a prototype software called “Decision Support System of IVF – Embryo transfer strategy determination” is developed, with the user interface shown in Figure 4.


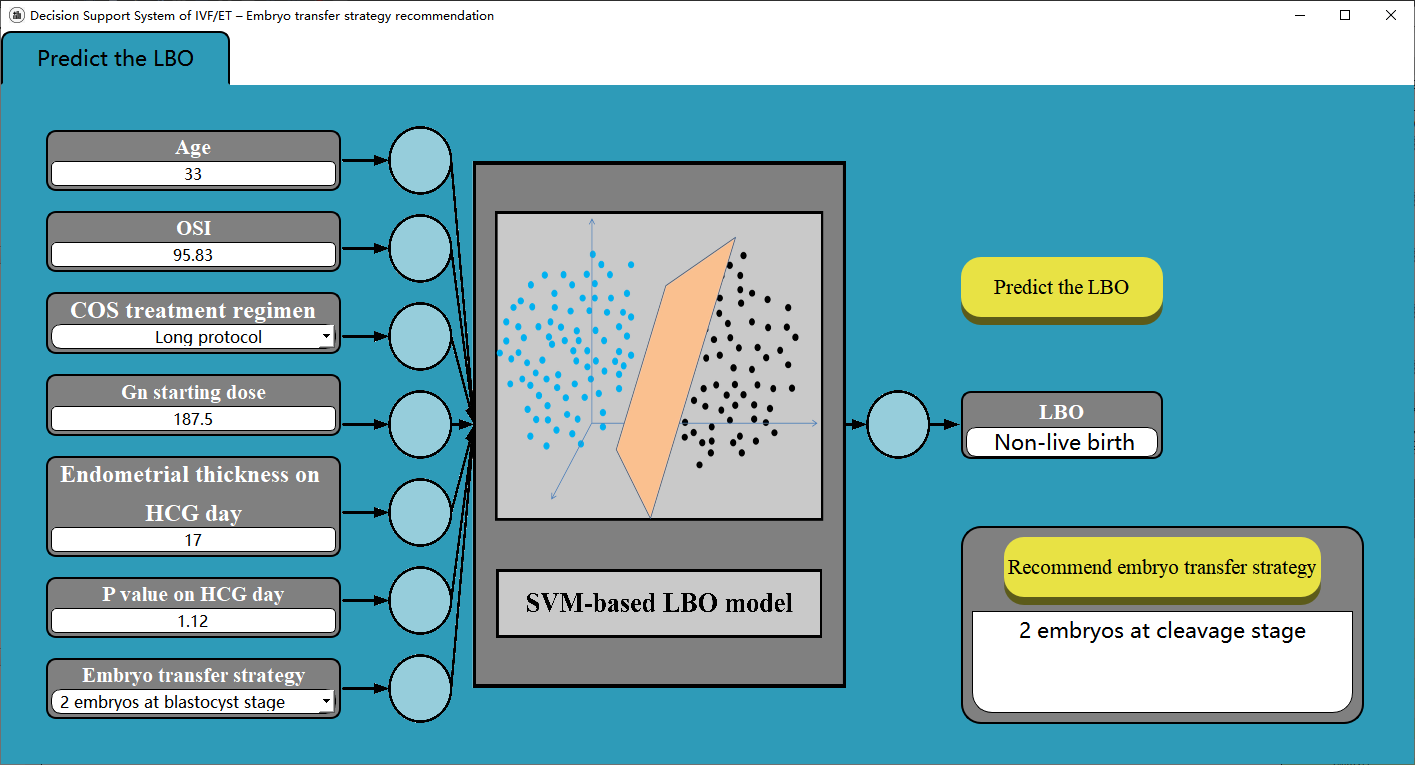


**Figure 4** Prototype software - Decision Support System of IVF – Embryo transfer strategy determination. For each patient, the user manually inputs the values of 7 influencing factors on the left side of the software and clicks the "Predict the LBO" button on the right to call the SVM-based LBO model embedded in the software. The predicted result will show in the dialog box of “LBO”. By clicking the “Recommend embryo transfer strategy” button, the strategy that has good LBO will be recommended in the dialog box. The information displayed on the software interface in Figure 4 is the clinical information of Patient # 48 and its corresponding predicted LBO, for which the predicted LBO is “Non-live birth” and the recommended strategy is “2 embryos at cleavage stage”.

The predictive performance of the established SVM-based LBO model was verified in actual clinical practices. For each of the new 82 patients, the information of 7 impact factors (i.e., OSI, COS treatment regimen, Gn starting dose, endometrial thickness on HCG day, P value on HCG day, and embryo transfer strategy) was taken as the input of the software (shown in Figure 4), and the SVM-based LBO model embedded in the software is used for prediction.

The overall prediction results (i.e., ROC) of 82 patients are shown in Figure 5, with the evaluation indicators of the prediction: AUC=0.862, precision=90.57%, sensitivity=75.00%, accuracy=74.39%, F1 score = 82.05%. Prediction results prove that the proposed model has good prediction performance, which further verifies the effectiveness of the model.


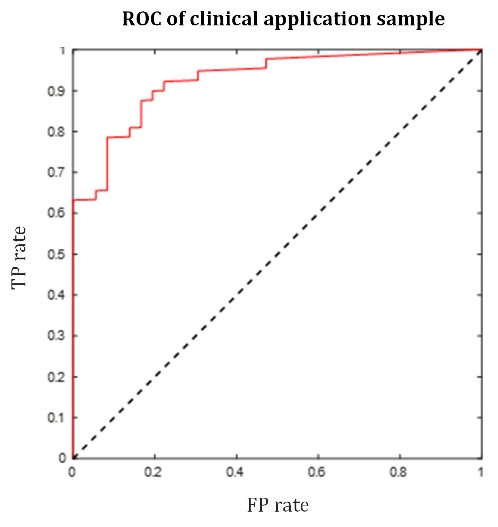


**Figure 5** ROC of the proposed model on the clinical application sample.

Also, the calibration plot and decision curve analysis (DCA) of the proposed model toward the clinical application sample are shown in Figure 6. Both the apparent and bias-corrected curves are pretty close to the ideal line (Figure 6(A)), and by utilizing our model, the net benefit could constantly be improved significantly for any threshold probability (Figure 6(B)); therefore, the effectiveness of the model in terms of prediction the LOB and clinical application is further validated.


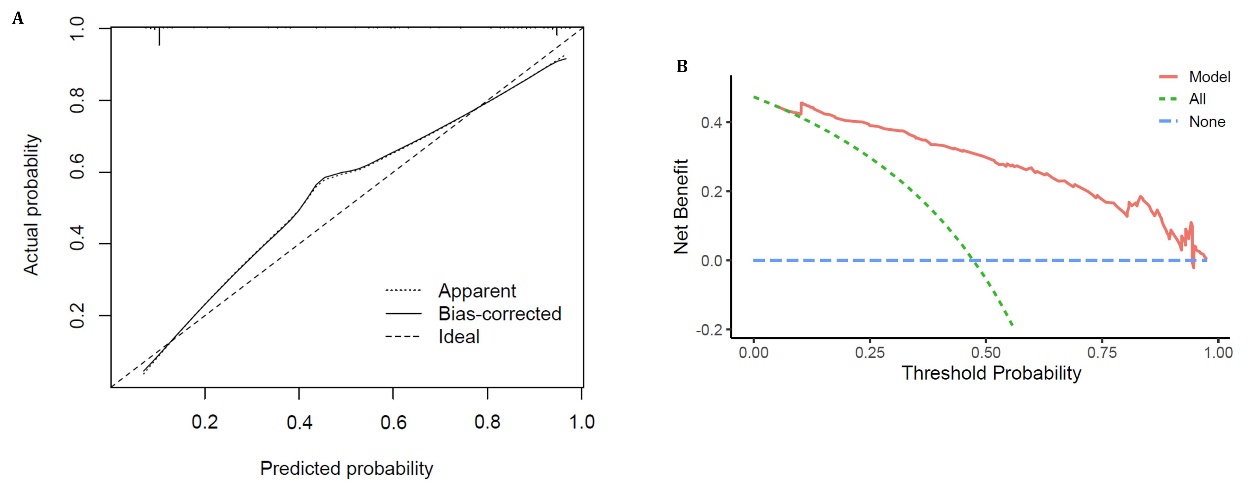


**Figure 6** (A) Calibration plot of the proposed model; (B) DCA curve of the proposed model

**3.4.2** **Clinical application of the proposed model in recommending the embryo transfer strategy**

In addition to the prediction of LBOs, the established model is utilized to guide the clinical practice, e.g., achieving optimal embryo transfer strategy in the IVF cycle. The process of customizing the embryo transfer strategy involves two steps:

1. Given six basic indicators of the patients (Age, OSI, COS treatment regimen, Gn starting dose, endometrial thickness on HCG day, and P value on HCG day), the corresponding LBO is predicted for the four embryo transfer strategies (i.e., Strategy-1: 1 embryo at cleavage stage; Strategy-2: 2 embryos at cleavage stage; Strategy-3: 1 embryo at blastocyst stage; Strategy-4: 2 embryos at blastocyst stage).
2. Among four predicted LBOs, the one with the good outcome (i.e., live birth) will be selected as the optimal strategy, which will be further used to assist clinicians in making the final decision on embryo transfer.

The recommended embryo transfer strategy based on our model for the 82 patients is shown in Figure 7. As can be seen from the figure, there may be several different alternatives for the same patient, and all of which have the predicted live birth. For example, both Strategy-2 and Strategy-4 can give Patient #4 live birth; Patient #20 has even more options: Strategy-2, Strategy-3, and Strategy-4.


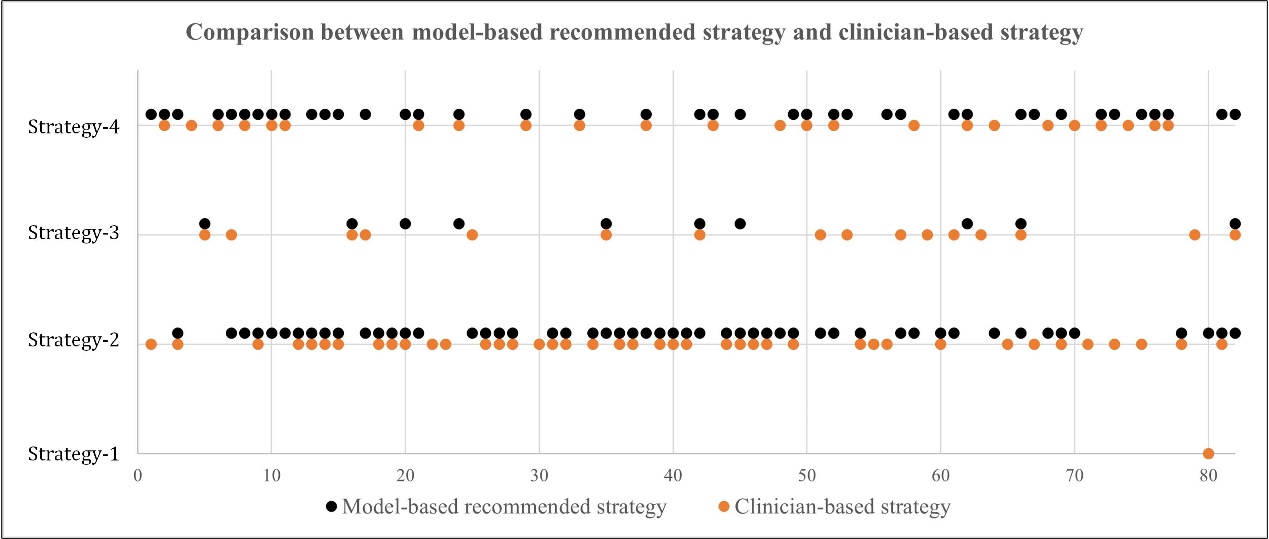


**Figure 7** Comparison between model-based recommended strategy and clinician-based strategy (In the figure, the horizontal axis represents the patient ID., and the vertical axis represents the embryo transfer strategy)

Compared with a clinician-based embryo transfer strategy that relies on the experience of clinicians, the recommended strategy based on the proposed model may have better LBOs for some patients. For example, for Patient #48 shown in Figure 4: the clinician-based decision is Strategy-4, and the outcome is non-live birth; however, the recommended result from our model is Strategy-2 (as shown in Figure 7), meaning that, by choosing Strategy-2 instead of Strategy-4, Patient #48 could have a greater likelihood of live birth. Similarly, for Patient # 53, if Strategy-4 is adopted instead of Strategy-3, it has a greater probability of obtaining a live birth.

**4. Discussion**

This study constructed a novel LBO prediction model based on the retrospective clinical data from 1405 patients. Before constructing the model, univariate and multivariate analysis identified 7 statistically significant influencing factors out of 16: Age, OSI, COS treatment regimen, Gn starting dose, endometrial thickness on HCG day, P value on HCG day and embryo transfer strategy. By taking the 7 screened factors as inputs and the LBO as outputs, two machine learning models, i.e., the ANN-based LBO model and the SVM-based LBO model, were established. By comparing the evaluation indexes of the two models, the SVM-based LBO model demonstrates superior modeling performance, with precision: 77.50% (test set) ~86.41% (training set), sensitivity: 75.58%~76.86%, accuracy: 80.43%~84.78%, F1 score: 77.18%~80.63%, AUC: 0.854~0.912. Therefore, the SVM-based LBO model is selected as the final model for predicting the LBO. Compared with the prevailing research methods, the model proposed in this study demonstrates significantly improved predictive performance.

Our proposed model demonstrates that female age is a significant predictor of LBO. As women age, their ovarian reserve capacity and reactivity decrease, leading to a reduction in the number of oocytes and embryos acquired [2, 3, 6], decreased fertilization ability of eggs and developmental potential of embryos, and an increased proportion of abortion and abnormal birth [7, 8]. In clinical practice, it is advisable for older women to promptly arrange a pregnancy plan and actively engage in pregnancy assistance intervention.

Our finding in Section 3 also revealed that the Gn starting dose is an essential factor affecting the LBO. The individualization of Gn starting dose is a standard clinical practice during COS in patients undergoing ART treatment [9], and it is determined based on the patient's age, AMH, AFC, bFSH, BMI, etc [10]. A small amount of Gn starting dose will lead to insufficient follicle recruitment. However, a large amount will lead to excessive follicle recruitment [11], resulting in an increased incidence of OHSS, and a rise in the progesterone level during COS, ultimately leading to an increase in the cancellation rate of fresh cycle transfer or a decrease in pregnancy rate [12] due to asynchronous endometrial development.

In our proposed model, we have established a robust correlation between the Ovarian Sensitivity Index (OSI), a composite measure of ovarian response[13, 14], and the LBO in patients undergoing IVF treatment for the first time. While previous studies have relied on indicators such as the number of retrieved oocytes, which are often used to reflect ovarian responsiveness, to predict LBO [15], clinical observations have revealed that patients with high ovarian response can still achieve favorable LBO outcomes even with minimal Gn dosage and a smaller quantity of retrieved oocytes [16]. On the contrary, for patients with poor ovarian response (POR), even with increased doses and duration of Gn stimulation and a normal number of oocytes obtained, the live birth rate could still remain low [17, 18]. The concept of OSI serves as a superior measure of ovarian responsiveness to Gn stimulation [14, 19]. In this study, we chose OSI as an indicator due to its comprehensive reflection of both the total dose of Gn and the ovarian response. It allows for simplification in inputting the proposed model without compromising modeling performance.

Results in Section 3 also indicate that the COS treatment regimen is a crucial determinant of LBO. In contrast to agonists, antagonists lack the "flare-up" effect and can suppress Gn within a few hours without causing excessive pituitary gland suppression, thereby reducing the required dose and duration of Gn and significantly lowering the incidence of OHSS [20, 21]. Nevertheless, numerous studies have demonstrated that the live birth outcome associated with GnRH antagonist regimens is inferior to that of GnRH agonist long regimens [22-24]. In our study, a total of 353 patients were treated with GnRH antagonist regimens, among which only 94 achieved live birth, with a live birth rate of 26.63%, which was much lower than the 55.24% of the GnRH agonist long regimens and the 43.05% of the Ultra-long GnRH agonist regimens. This phenomenon may be attributed to reduced endometrial receptivity in infertile women undergoing GnRH antagonist regimens, leading to a decreased embryo implantation rate [25].

Scholars have suggested establishing a threshold for the P value on HCG day at 1.5-2.0ng/mL [26] due to the potential risk of fresh transplant pregnancy failure associated with higher values [27]. Furthermore, studies have indicated that an increase in P value on HCG day may lead to abnormal expression of endometrial embryo implantation proteins (vascular endothelial growth factor and placental expression factor) and differences in epigenetic profiles, ultimately leading to asynchronous development of the embryo and endometrium and adverse pregnancy outcomes [28]. Elevated P value on HCG day has also been linked to reduced oocyte and embryo quality, leading to lower rates of excellent embryos and cumulative live birth rates [29]. These findings are consistent with the negative partial regression coefficient of P value on HCG day (i.e., B=-0.310) presented in Table 2.

The appropriate endometrial thickness on HCG day is crucial for successful embryo implantation. Some studies have reported that no pregnancy occurs when endometrial thickness is less than 5mm [30]. Additionally, when the endometrial thickness falls below 7mm [31, 32] or exceeds 16mm, it is not conducive to embryo transfer and implantation, which results in a low clinical pregnancy rate [33]. Currently, endometrial thickness of more than 7mm is considered the conventional lower limit for embryo transfer. Our study suggests that endometrial thickness on HCG day should be regarded as an important index before embryo transfer; endometrial thickness should be adjusted to the ideal thickness before transfer to improve the live birth rate.

Embryos are usually transferred into the womb during the cleavage stage (2-3 days after fertilization) in early times. Prolongation of embryo culture time in vitro to blastocyst stage (5-6 days after fertilization) has been widely employed recently, with the benefits of screening high-quality embryos and keeping the embryo development stage relatively synchronous with the endometrium [34]. Studies have reported that blastocyst transfer yields a higher clinical pregnancy rate and birth rate than cleavage embryos when an equivalent number of embryos are transferred [35-37]. The data in Table 1 also demonstrates that the live birth rate following blastocyst transfer is significantly higher than that of embryo transfers at the cleavage stage (1 embryo transfer: 22.42% vs. 15.38%; 2 embryo transfer: 54.34% vs. 46.34%). Two cleaved embryos and one or two blastocyst embryos were independent promotors of clinical live birth compared with one cleaved embryo. It is important to note that the outcome of IVF live birth is not only related to the timing and number of embryos transferred but also the quality of the embryos [38]. However, due to the inherent subjectivity in embryo quality rating and the lack of widespread adoption of time-lapse technology in IVF centers, IVF center rating is not entirely consistent, so we did not include factors of embryo quality rating in this study. This is an aspect that will be explored in future research, utilizing a machine learning algorithm to assess the impact of embryo quality on LBOs. Overall, our findings highlight the significant influence of both stage and number of embryos transferred on the outcome of live birth.

The findings in Section 3.4 indicate that the recommended strategy derived from the proposed model may yield superior LBOs for some patients compared to the clinician-based embryo transfer approach. These results have potential implications for informing evidence-based decision-making in IVF clinical practice. Due to the traditional preference for transferring 2 embryos in IVF-ET treatment, the historical data utilized to model the LBO also predominantly supports this strategy, leading to a tendency towards a potential transplant strategy of 2 embryos (the clinical LBO of Strategy-3 is significantly higher than that of Strategy-1 in section 3.4.2 ). However, scholars believe higher LBOs can be achieved with a single blastocyst transferred (i.e., Strategy-3), and this strategy is becoming the mainstream method of embryo transfer for its decreased multiple pregnancies rate and OHSS risk and increased cumulative pregnancy rate. As the practice of single blastocyst transfer gradually becomes prevalent in current and forthcoming data, the expertise and knowledge encompassed in the newly established model will be continuously updated to align with this mainstream embryo transfer strategy, thereby enhancing the recommended outcomes.

The proposed LBO prediction model in this study is developed by leveraging clinical big data through machine learning techniques. The model considers a comprehensive range of factors, including basic clinical characteristics, COS treatment regimen, OSI, P value on HCG day, endometrial thickness on HCG day, and embryo transfer-related indicators to capture the critical processes in the IVF cycle. Based on the established prediction model, it is possible to forecast LBO by iteratively selecting different embryo transfer strategies and ultimately identifying the optimal strategy based on expected outcomes.

In addition to the recommendation of embryo transfer strategy, the proposed model is also suitable for decision-making on other influencing factors of the LBO model. For example, the model can calculate the endometrial thickness interval, from which the expected live birth situation can be achieved. In this way, whether or not to perform embryo transfer can be determined based on actual endometrial thickness and the prediction LBO. This will be another future work.

**5. Conclusions**

We have developed an SVM-based model that can accurately predict the LBO of the IVF, as measured by the indicators of precision, accuracy, sensitivity, and F1 score. This model has been successfully applied in clinical practice to provide precise LBO predictions and as a reliable and scientific tool for guiding decision-making in IVF treatment. For example, it can recommend embryo transfer strategies, optimize COS treatment regimens, and determine the ideal endometrial thickness interval for future embryo transfers. It is of great significance in making treatment decisions, alleviating patients’ psychological burden, and promoting patient compliance throughout the IVF treatment cycle.

**List of abbreviations**

ART: assisted reproductive technology; IVF-ET: in vitro fertilization and embryo transfer; LBO: live birth outcome; AMH: anti-Müllerian hormone; AFC: antral follicle count; BMI: body mass index; Gn: gonadotropin; ICSI: intracytoplasmic sperm injection; LH: luteinizing hormone; E_2_: estradiol; bFSH: basal follicle stimulating hormone; AFC: antral follicle count; P: progesterone; PPOS: progestin-primed ovarian stimulation; COS: controlled ovarian stimulation; HCG: human chorionic gonadotrophin; OSI: ovarian sensitivity index; 2PN: pronuclei; ANN: artificial neural network; SVM: supporting vector machine; ROC: receiver operating characteristic; TP: true positive; FP: False Positive; TN: True Negative; FN: False Negative; DCA: decision curve analysis (DCA); OHSS: ovarian hyperstimulation syndrome.

**Ethics approval and consent to participate**

The study was conducted according to the guidelines of the Declaration of Helsinki, and approved by the Medical Ethics Committee of Renmin Hospital of Wuhan University (Approval number WDRY2019-K077, 26 November 2019). Informed consent has been obtained from all the patients involved in this research.

**Consent for publication**

Not applicable

**Availability of data and materials**

The datasets used and/or analyzed during the current study are available from the corresponding author upon reasonable request.

**Competing interests**

The authors declare that they have no competing interests

**Funding**

This work was supported by the National Natural Science Foundation of China (grant number 82001642).

**Authors' contributions**

Liu Liu completed the first draft; Fujin Shen, and Liangfei Ao conceived and designed this article; Fujin Shen, Jiao Chen and Liangfei Ao revised the manuscript draft; Liu Liu and Hua Liang collected the clinical data and conducted preliminary analysis; Liu Liu established the model and analyzed the results. Hua Liang and Jing Yang coordinated clinical data collection and statistics. All authors read and approved the final manuscript.

**Acknowledgments**

We appreciate Dr. Wei Li, Dr. Yang Li and Mr. Zhe Yang for providing valuable suggestions for drafting this manuscript.

**Reference**

1. McLernon DJ, Steyerberg EW, Te Velde ER, Lee AJ, Bhattacharya S: **Predicting the chances of a live birth after one or more complete cycles of in vitro fertilisation: population based study of linked cycle data from 113 873 women.** *bmj* 2016, **355**.

2. Dhillon R, McLernon D, Smith P, Fishel S, Dowell K, Deeks J, Bhattacharya S, Coomarasamy A: **Predicting the chance of live birth for women undergoing IVF: a novel pretreatment counselling tool.** *Human Reproduction* 2016, **31:**84-92.

3. Van Loendersloot L, Van Wely M, Repping S, Bossuyt P, Van Der Veen F: **Individualized decision-making in IVF: calculating the chances of pregnancy.** *Human Reproduction* 2013, **28:**2972-2980.

4. Metello JL, Tomás C, Ferreira P: **Can we predict the IVF/ICSI live birth rate?** *JBRA Assisted Reproduction* 2019, **23:**402.

5. Wen M, Wu F, Du J, Lv H, Lu Q, Hu Z, Diao F, Ling X, Tan J, Jin G: **Prediction of live birth probability after in vitro fertilization and intracytoplasmic sperm injection treatment: A multi‐center retrospective study in Chinese population.** *Journal of Obstetrics and Gynaecology Research* 2021, **47:**1126-1133.

6. Lu X, Liu Y, Xu J, Cao X, Zhang D, Liu M, Liu S, Dong X, Shi H: **Mitochondrial dysfunction in cumulus cells is related to decreased reproductive capacity in advanced-age women.** *Fertil Steril* 2022, **118:**393-404.

7. Ferraretti A, La Marca A, Fauser B, Tarlatzis B, Nargund G, Gianaroli L, Definition EwgoPOR: **ESHRE consensus on the definition of ‘poor response'to ovarian stimulation for in vitro fertilization: the Bologna criteria.** *Human reproduction* 2011, **26:**1616-1624.

8. De Bruin J, Dorland M, Spek E, Posthuma G, Van Haaften M, Looman C, Te Velde E: **Age-related changes in the ultrastructure of the resting follicle pool in human ovaries.** *Biology of reproduction* 2004, **70:**419-424.

9. Fatemi H, Bilger W, Denis D, Griesinger G, La Marca A, Longobardi S, Mahony M, Yin X, D’Hooghe T: **Dose adjustment of follicle-stimulating hormone (FSH) during ovarian stimulation as part of medically-assisted reproduction in clinical studies: a systematic review covering 10 years (2007–2017).** *Reproductive Biology and Endocrinology* 2021, **19:**68.

10. Chen M-X, Meng X-Q, Zhong Z-H, Tang X-J, Li T, Feng Q, Adu-Gyamfi EA, Jia Y, Lv X-Y, Geng L-H: **An individualized recommendation for controlled ovary stimulation protocol in women who received the GnRH agonist long-acting protocol or the GnRH antagonist protocol: a retrospective cohort study.** *Frontiers in endocrinology* 2022, **13:**899000.

11. Holt-Kentwell A, Ghosh J, Devall A, Coomarasamy A, Dhillon-Smith RK: **Evaluating interventions and adjuncts to optimize pregnancy outcomes in subfertile women: an overview review.** *Human reproduction update* 2022, **28:**583-600.

12. Li Y, Duan Y, Yuan X, Cai B, Xu Y, Yuan Y: **A novel nomogram for individualized gonadotropin starting dose in GnRH antagonist protocol.** *Frontiers in Endocrinology* 2021, **12:**688654.

13. He Y, Liu L, Yao F, Sun C, Meng M, Lan Y, Yin C, Sun X: **Assisted reproductive technology and interactions between serum basal FSH/LH and ovarian sensitivity index.** *Front Endocrinol (Lausanne)* 2023, **14:**1086924.

14. Li HWR, Lee VCY, Ho PC, Ng EHY: **Ovarian sensitivity index is a better measure of ovarian responsiveness to gonadotrophin stimulation than the number of oocytes during in-vitro fertilization treatment.** *Journal of assisted reproduction and genetics* 2014, **31:**199-203.

15. Liu L, Shen F, Liang H, Yang Z, Yang J, Chen J: **Machine learning-based modeling of ovarian response and the quantitative evaluation of comprehensive impact features.** *Diagnostics* 2022, **12:**492.

16. Verberg M, Eijkemans M, Macklon N, Heijnen E, Baart E, Hohmann F, Fauser B, Broekmans F: **The clinical significance of the retrieval of a low number of oocytes following mild ovarian stimulation for IVF: a meta-analysis.** *Human reproduction update* 2009, **15:**5-12.

17. Saldeen P, Källen K, Sundström P: **The probability of successful IVF outcome after poor ovarian response.** *Acta obstetricia et gynecologica Scandinavica* 2007, **86:**457-461.

18. Hua L, Zhe Y, Jing Y, Fujin S, Jiao C, Liu L: **Prediction model of gonadotropin starting dose and its clinical application in controlled ovarian stimulation.** *BMC Pregnancy and Childbirth* 2022, **22:**1-14.

19. Huber M, Hadziosmanovic N, Berglund L, Holte J: **Using the ovarian sensitivity index to define poor, normal, and high response after controlled ovarian hyperstimulation in the long gonadotropin-releasing hormone-agonist protocol: suggestions for a new principle to solve an old problem.** *Fertility and sterility* 2013, **100:**1270-1276. e1273.

20. Huirne J, Homburg R, Lambalk C: **Are GnRH antagonists comparable to agonists for use in IVF?** *Human Reproduction* 2007, **22:**2805-2813.

21. Venetis CA, Storr A, Chua SJ, Mol BW, Longobardi S, Yin X, D'Hooghe T: **What is the optimal GnRH antagonist protocol for ovarian stimulation during ART treatment? A systematic review and network meta-analysis.** *Hum Reprod Update* 2023, **29:**307-326.

22. Al-Inany HG, Youssef MA, Aboulghar M, Broekmans F, Sterrenburg M, Smit J, Abou-Setta AM: **GnRH antagonists are safer than agonists: an update of a Cochrane review.** *Human reproduction update* 2011, **17:**435-435.

23. Rabinson J, Meltcer S, Zohav E, Gemer O, Anteby EY, Orvieto R: **GnRH agonist versus GnRH antagonist in ovarian stimulation: the influence of body mass index on in vitro fertilization outcome.** *Fertility and sterility* 2008, **89:**472-474.

24. Kuan KKW, Omoseni S, Tello JA: **Comparing ART outcomes in women with endometriosis after GnRH agonist versus GnRH antagonist ovarian stimulation: a systematic review.** *Ther Adv Endocrinol Metab* 2023, **14:**20420188231173325.

25. Rackow BW, Kliman HJ, Taylor HS: **GnRH antagonists may affect endometrial receptivity.** *Fertility and sterility* 2008, **89:**1234-1239.

26. Hill MJ, Healy MW, Richter KS, Parikh T, Devine K, DeCherney AH, Levy M, Widra E, Patounakis G: **Defining thresholds for abnormal premature progesterone levels during ovarian stimulation for assisted reproduction technologies.** *Fertility and sterility* 2018, **110:**671-679. e672.

27. Yang Y, Liu B, Wu G, Yang J: **Exploration of the value of progesterone and progesterone/estradiol ratio on the hCG trigger day in predicting pregnancy outcomes of PCOS patients undergoing IVF/ICSI: a retrospective cohort study.** *Reprod Biol Endocrinol* 2021, **19:**184.

28. Drakopoulos P, Racca A, Errázuriz J, De Vos M, Tournaye H, Blockeel C, Pluchino N, Santos-Ribeiro S: **The role of progesterone elevation in IVF.** *Reproductive biology* 2019, **19:**1-5.

29. Racca A, Santos-Ribeiro S, De Munck N, Mackens S, Drakopoulos P, Camus M, Verheyen G, Tournaye H, Blockeel C: **Impact of late-follicular phase elevated serum progesterone on cumulative live birth rates: is there a deleterious effect on embryo quality?** *Human Reproduction* 2018, **33:**860-868.

30. Abdalla H, Brooks A, Johnson M, Kirkland A, Thomas A, Studd J: **Endometrial thickness: a predictor of implantation in ovum recipients?** *Human Reproduction* 1994, **9:**363-365.

31. Mahutte N, Hartman M, Meng L, Lanes A, Luo Z-C, Liu KE: **Optimal endometrial thickness in fresh and frozen-thaw in vitro fertilization cycles: an analysis of live birth rates from 96,000 autologous embryo transfers.** *Fertility and sterility* 2022, **117:**792-800.

32. Song L, Bu Z, Sun Y: **Endometrial thickness and early pregnancy complications after frozen-thawed embryo transfers.** *Frontiers in Endocrinology* 2023, **14:**1066922.

33. Jung Y, Kim Y, Kim M, Park I, Yoo Y, Jo J: **Endometrial injury may promote implantation in patients with increased endometrial thickness on the day of hCG administration.** *Fertility and Sterility* 2013, **100:**S388.

34. Lee AM, Connell MT, Csokmay JM, Styer AK: **Elective single embryo transfer-the power of one.** *Contraception and Reproductive Medicine* 2016, **1:**1-7.

35. Glujovsky D, Farquhar C, Retamar AMQ, Sedo CRA, Blake D: **Cleavage stage versus blastocyst stage embryo transfer in assisted reproductive technology.** *Cochrane database of systematic reviews* 2016.

36. Dar S, Lazer T, Shah P, Librach C: **Neonatal outcomes among singleton births after blastocyst versus cleavage stage embryo transfer: a systematic review and meta-analysis.** *Human reproduction update* 2014, **20:**439-448.

37. Glujovsky D, Quinteiro Retamar AM, Alvarez Sedo CR, Ciapponi A, Cornelisse S, Blake D: **Cleavage-stage versus blastocyst-stage embryo transfer in assisted reproductive technology.** *Cochrane Database Syst Rev* 2022, **5:**Cd002118.

38. Dupont C, Hafhouf E, Sermondade N, Sellam O, Herbemont C, Boujenah J, Faure C, Levy R, Poncelet C, Hugues J-N: **Delivery rates after elective single cryopreserved embryo transfer related to embryo survival.** *European Journal of Obstetrics & Gynecology and Reproductive Biology* 2015, **188:**6-11.

1. # These two authors contribute equally to this research [↑](#footnote-ref-1)
2. [↑](#footnote-ref-2)
